# A case report of AQP4-IgG-seropositive refractory neuromyelitis optica spectrum disorder patient with Sjögren’s syndrome and pancytopenia treated with inebilizumab

**DOI:** 10.3389/fneur.2024.1371515

**Published:** 2024-06-05

**Authors:** Shasha Li, Yuting Gao, Yang He, Zhaoxu Zhang

**Affiliations:** ^1^Graduate School, Beijing University of Chinese Medicine, Beijing, China; ^2^Department of Neurology, The First Hospital of Hebei Medical University, Shijiazhuang, China; ^3^Department of Neurology, Peking University People’s Hospital, Beijing, China

**Keywords:** neuromyelitis optica spectrum disorder, Sjögren’s syndrome, pancytopenia, inebilizumab, aquaporin-4, case report

## Abstract

Patients with neuromyelitis optica spectrum disorder (NMOSD) coexisting with both Sjögren’s syndrome (SS) and pancytopenia are exceptionally rare. There is no study on the treatment of such patients. We presented a case of AQP4-IgG seropositive refractory NMOSD patient combined with SS and pancytopenia with significant response to inebilizumab. In 2017 the 49-year-old female patient was diagnosed with SS and pancytopenia without any treatment. In August 2022, she had a sudden onset of lower limbs weakness, manifested as inability to walk, accompanied by urinary incontinence. After receiving methylprednisolone and cyclophosphamide, she regained the ability to walk. In February 2023, she suffered from weakness of both lower limbs again and paralyzed in bed, accompanied by retention of urine and stool, and loss of vision in both eyes. After receiving methylprednisolone and three plasmapheresis, the condition did not further worsen, but there was no remission. In March 2023, the patient was admitted to our hospital and was formally diagnosed with AQP4-IgG seropositive NMOSD combined with SS and pancytopenia. After receiving two 300 mg injections of inebilizumab, not only the symptoms of NMOSD improved significantly, but also the symptoms of concurrent SS and pancytopenia. In the cases of AQP4-IgG seropositive NMOSD who have recurrent episodes and are comorbid with other autoimmune disorders, inebilizumab may be a good choice.

## 1 Introduction

Neuromyelitis optica spectrum disorder (NMOSD) is a rare and severe autoimmune demyelinating disease of the central nervous system (CNS). It mainly involves the optic nerve and spinal cord, causing paralysis, visual impairment, and encephalopathy. In recent decades, NMOSD has been recognized as a distinct entity from multiple sclerosis (MS). The identification of a pathogenic autoantibody targeting aquaporin-4 (AQP4) has served to differentiate NMOSD from MS, establishing it as a separate disease (1). AQP4 is expressed highest in astrocytes. AQP4-Immunoglobulin G (IgG) can lead to astrocyte damage and inflammation; axonal injury and demyelination are the secondary phenomenon resulting from the highly destructive pathogenic process (2). NMOSD follows a relapse-remission course, marked by severe recurrent episodes and the accumulation of permanent disability, eventually leading to the death of approximately one-third of NMOSD patients (3).

Approximately one in four patients with AQP4-IgG positive NMOSD have another coexisting autoimmune diseases, with systemic lupus erythematosus (SLE), rheumatoid arthritis (RA), and Sjögren’s syndrome (SS) being the most common complications (4). SS is a systemic chronic autoimmune disease that mainly affects women of middle age (5). The condition may evolve from an asymptomatic, indolent course, with glandular involvement, to extra-glandular systemic manifestations, leading to extremely pleomorphic clinical symptoms. Nervous system involvement occurs in up to 20% of patients with SS (6). In a cohort of 1,010 cases diagnosed with SS, 2% had CNS disease, including neuromyelitis optica (NMO) (7). The incidence of myelitis in patients with SS is unknown, but estimated at less than 1% (8).

Currently, there are no guidelines and recommendations for the treatment of NMOSD combined with SS worldwide. Inebilizumab has been approved for the treatment of adult AQP4-IgG seropositive NMOSD in many countries. This study presents for the first time a case of AQP4-IgG seropositive refractory NMOSD patient combined with SS and pancytopenia with significant response to inebilizumab.

## 2 Case presentation

In 2017 the 49-year-old female patient was diagnosed with SS and pancytopenia, manifested by intermittent decrease in erythrocyte, leukocyte, and platelets, dry eyes and mouth, and anti-SSA antibody (+). She did not receive any treatment due to minimal impact on her daily life.

**Figure 1:**
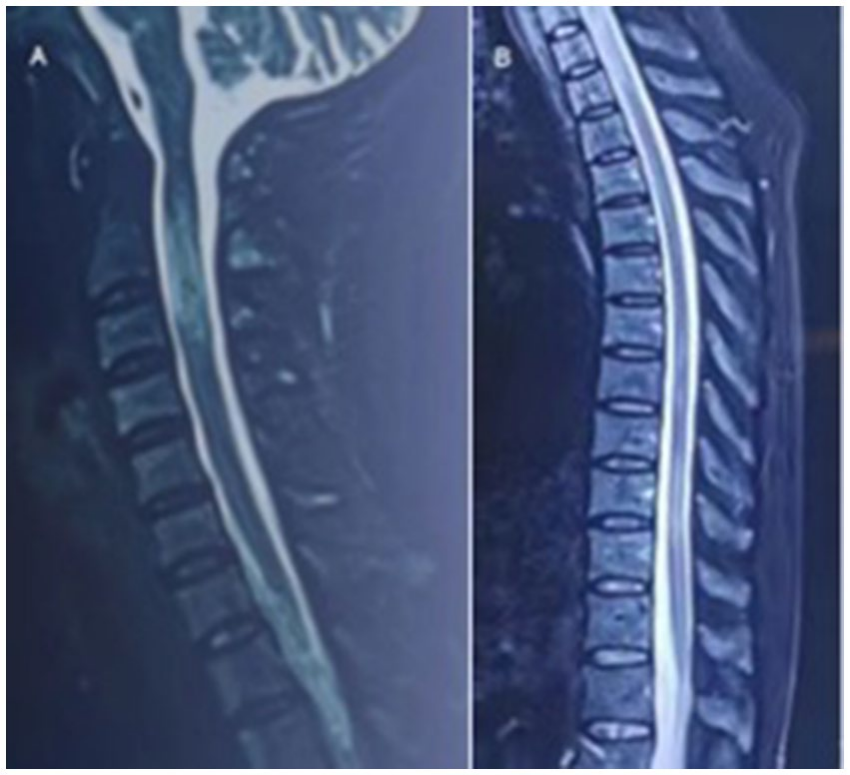
In September 2022, sagittal MRI of the cervical and thoracic vertebrae showed continuous central spinal cord hyperintensities.

In August 2022, she had a sudden onset of lower limbs weakness, manifested as inability to walk, accompanied by urinary incontinence. And she had needle like pain and itching all over her body, particularly in the neck and upper limbs. Afterwards, she went to hospital for treatments. After admission, sagittal MRI of the cervical and thoracic vertebrae showed continuous central spinal cord hyperintensities (Figure 1). After receiving high-dose intravenous methylprednisolone (IVMP) 1 g and one infusion of cyclophosphamide (Cy) 0.6 g treatment, she regained the ability to walk. After discharge, IVMP was changed to oral administration and gradually decreased. She discontinued using methylprednisolone after 3 months.

In February 2023, the patient suffered from weakness of both lower limbs again and paralyzed in bed unable to lift both lower limbs off the bed surface, accompanied by retention of urine and stool. Sensation at the level of chest nipple was lost. At the same time, there was a loss of vision in both eyes. From February 16th to February 20th, she received IVMP 1 g for three consecutive days, and on February 21st, oral hormone of 60 mg was applied. However, her symptoms continued to worsen, sensory plane rose to the sternal stalk segment, and both lower limbs were completely paralyzed with grade I muscle strength. Then, three plasmapheresis (PLEX) were performed on February 24th, February 27th, and March 1st. After that, the condition did not further worsen, but there was no remission and systemic edema appeared.

In March 2023, laboratory tests showed positive AQP4-IgG in serum and cerebrospinal fluid (CSF), as well as anti-nuclear antibody (+), anti-SSA antibody (+), anti-Ro-52 antibody (+), white blood cell count of 3.57*10^9^/L (lymphocyte of 3%), hemoglobin level of 78 g/L, and platelet count of 21*10^9^/L in peripheral blood. Schirmer Test ≤ 5 mm/5 min. Natural saliva flow rate ≤ 0.1 mL/min. The patient was formally diagnosed with AQP4-IgG seropositive NMOSD combined with SS at other hospital.

In March 17th, 2023, the patient was admitted to our hospital. She had grade I muscle strength in both lower limbs, positive pathologic signs bilaterally, retention of urine and stool, significant visual impairment, dry mouth, dry eyes, and dental caries. She was treated with sucrose iron, folic acid and mecobalamin to supplement hematopoietic substances, albumin to promote bone marrow hematopoiesis, Amino-Polypeptide tablets to elevate platelets, and prednisone acetate 60 mg orally for 3 days, along with diuretic, anticoagulant, and anxiety-relieving treatment. Laboratory tests upon admission showed a hemoglobin level of 78 g/L, platelet count of 24*10^9^/L, white blood cell count of 9.5*10^9^/L (lymphocyte of 8%), B-lymphocyte absolute count of 47.57/μL, serum AQP4-IgG titer of 1:100, anti-nuclear antibody titer of 1:160, anti-SSA antibody (+), and anti-Ro-52 antibody (+). On March 22nd and April 13th, the patient received 300 mg injections of inebilizumab, respectively. By April 13th, she demonstrated grade III muscle strength in both lower limbs and positive pathologic signs bilaterally, relieved retention of urine and stool, gradual improvement in vision, persistent dry mouth, dry eyes, and dental caries symptoms. Laboratory results showed a hemoglobin level of 101 g/L, platelet count of 56*10^9^/L, white blood cell count of 4*10^9^/L (lymphocyte of 22%), B-lymphocyte absolute count of 42/μL, serum AQP4-IgG titer of 1:100, anti-nuclear antibody titer of 1:80, and anti-SSA antibody (+). On May 6th, the patient exhibited grade IV+ muscle strength in both lower limbs and negative pathologic signs bilaterally, her vision was recovered completely, and there was a relief in dry mouth and dry eyes and no retention of urine and stool. Peripheral blood tests showed a hemoglobin level of 116 g/L, platelet count of 56*10^9^/L, white blood cell count of 3.5*10^9^/L (lymphocyte 33%), B-lymphocyte absolute count of 3/μL, serum AQP4-IgG titer of 1:10, anti-nuclear antibody titer of 1:80, and anti-SSA antibody (+) (Table 1).

**Table 1:** Dynamics of the symptoms, signs, and laboratory findings related to neuromyelitis optica spectrum disorder, Sjögren’s Syndrome, and pancytopenia.

Neuromyelitis optica spectrum disorder	B-lymphocyte		–	–	–	–	–	47.57/μL	42/μL	3/μL
	Serum AQP4-IgG titer		–	–	–	–	–	1:100	1:100	1:10
	Systemic edema		(−)	(−)	(−)	(−)	(+)	(−)	(−)	(−)
	Visual impairment		(−)	(−)	(+)	(+)	(+)	(+)	Relieved	Recovered completely
	Sensation lost		(−)	(−)	Chest nipple level	Sternal stalk segment	Sternal stalk segment	(−)	(−)	(−)
	Pain and itching		Over the body	(−)	(−)	(−)	(−)	(−)	(−)	(−)
	Retention of urine and stool		(−)	(−)	(+)	(+)	(+)	(+)	Relieved	(−)
	Urinary incontinence		(+)	(−)	(−)	(−)	(−)	(−)	(−)	(−)
	Pathologic signs bilaterally		–	–	–	–	–	(+)	(+)	(−)
	Muscle strength in both lower limbs		IV−	IV+	II	I	I	I	III	IV+
Pancytopenia	Platelet	Intermittent decrease						24*10^9^/L	56*10^9^/L	56*10^9^/L
	Lymphocyte	–						8%	22%	33%
	White blood cell	Intermittent decrease						9.5*10^9^/L	4*10^9^/L	3.5*10^9^/L
	Hemoglobin	Intermittent decrease						78 g/L	101 g/L	116 g/L
Sjögren’s syndrome	Anti-Ro-52 antibody	–						(+)	(−)	(−)
	Anti-SSA antibody	(+)						(+)	(+)	(+)
	Anti-nuclear antibody	–						1:160	1:80	1:80
	Dental caries	(−)						(+)	(+)	(+)
	Dry eyes	(+)						(+)	(+)	Relieved
	Dry mouth	(+)						(+)	(+)	Relieved
	Treatment	–	No	After IVMP 1 g and Cy 0.6 g	No	After IVMP 3 g and oral hormone 60 mg	After three PLEX	Admitted to our hospital	After inebilizumab 600 mg	No
	Time	2017	August 2022	August 2022	February 2023	February 2023	March 2023	March 17th, 2023	April 13th, 2023	May 6th, 2023

AQP4-IgG, aquaporin-4-immunoglobulin G; IVMP, intravenous methylprednisolone; Cy, cyclophosphamide; PLEX, plasmapheresis.

## 3 Discussion

This case is a refractory NMOSD combined with SS and pancytopenia, which was prolonged and responded not well to conventional immunotherapy. After a comprehensive analysis of the condition, inebilizumab was selected. As a result, the symptoms, signs, and laboratory findings of NMO, SS, and pancytopenia were all improved. This case is reported for the first time and provides preliminary evidence for the application of inebilizumab for patients with NMOSD combined with other autoimmune diseases.

Although NMOSD may manifest at any age, the mean age of symptom onset is 40 years, a striking female predominance, at a ratio of approximately 9:1. The disorder predominantly affects the optic nerve and spinal cord. Up to 85% NMOSD patients exhibit longitudinally extensive transverse myelitis (LETM) (9). Optic neuritis (ON) in NMOSD often presents as a bilateral condition with severe visual impairment (10). Notably, 45% of AQP4-IgG-positive patients experience ON as an initial symptom, while 60% develop ON over the course of disease (11). AQP4-IgG seropositive is a critical factor in the clinical diagnosis of NMOSD, evident in over 80% of cases (12). Our patient, a 49-year-old woman, displayed MRI findings of continuous central spinal cord hyperintensities and clinical symptoms of ON and myelitis, along with positive AQP4-IgG in serum and CSF. According to the 2015 diagnostic criteria established by the International Panel for NMO Diagnosis (13), this case can be definitively diagnosed as AQP4-IgG positive NMOSD.

The main feature of SS is that the lacrimal and salivary glands are infiltrated by lymphocytes, causing Xerophthalmia and Xerostomia (14). Anti-nuclear antibody, detectable in the peripheral blood of up to 85% of SS patients, it considered too general a marker. More specific is the presence of Anti-Ro/SSA autoantibody, found in 33–74% of SS patients. In this patient, the presence of dry mouth and eyes, dental caries, anti-nuclear antibody (+), anti-SSA antibody (+), anti-Ro-52 antibody (+), along with a Schirmer Test ≤ 5 mm/5 min and a reduced natural saliva flow rate ≤ 0.1 mL/min, aligns with the 2016 the American College of Rheumatology and the European League Against Rheumatism Classification Criteria for Primary SS (15), confirming the diagnosis of SS. Notably, 7.7–12% of NMOSD patients also exhibit anti-SSA or anti-SSB antibodies (16). NMOSD combined with SS often presents with LETM and ON, tends to recur, and is associated with severe nerve damage and poor clinical prognosis (17). Interestingly, AQP4 expression is low in the salivary glands, while AQP5, which shares approximately 50% protein sequence identity with AQP4, is highly expressed (18). This shared sequence may prompt the immune system to target both the CNS and salivary glands, potentially explaining the coexistence of SS and NMOSD in this patient.

In some cases, patients present with pancytopenia without meeting the diagnostic criteria for known hematological or non-hematological diseases. However, they respond favorably to immunosuppressive treatments, such as corticosteroid and intravenous immunoglobulin. This condition is known as immune-related pancytopenia (IRP) (19), characterized by bone marrow damage inflicted by autoantibodies against hematopoietic cell (19). Both IgG and Immunoglobulin M antibody can cause intravascular hemolysis and severe anemia (20). Hematologic complications, including IRP are not uncommon in SS and can be life-threatening.

The primary treatment for acute attacks of NMOSD involves high-dose IVMP, administered at 1 g daily for three to seven consecutive days, followed by a gradual reduction using oral prednisolone over 2 weeks (21, 22). Cy typically used in treating cancers and immune-mediated inflammatory conditions, is also effective in early-stage autoimmune diseases where inflammation is more prominent than degenerative processes. Treatment regimens often involve intravenous pulse therapy with or without corticosteroids every 4–8 weeks (23). Several studies have demonstrated the effectiveness of Cy, either alone or in combination with other treatments, in managing NMOSD-related LETM (24, 25). In this case, the patient’s NMOSD-associated myelitis symptoms were initially managed with IVMP combined with Cy. However, about 6 months later, the myelitis symptoms recurred and worsened, accompanied by ON. NMOSD typically follows a relapsing course in about 90% of cases, with intervals ranging from months to years between attacks. The most frequent relapse types include ON and transverse myelitis, particularly prevalent in individuals over 30 years of age (26). Patients with AQP4-IgG positive who experience a myelitis episode have a 60% risk of another episode within 1 year (27).

The standard initial treatment for acute relapses of NMOSD typically involves high-dose corticosteroids, most commonly IVMP (21, 22). However, the effectiveness of IVMP monotherapy is relatively modest. In one study encompassing 693 NMOSD attacks, 16.2% of patients showed no improvement following IVMP treatment alone (28). Consequently, for many patients, the concurrent or immediate addition of PLEX is often necessary (21, 29). In fact, the combined use of PLEX/IVMP has demonstrated superior efficacy compared to monotherapy (30, 31). In the case of our patient, the limited response to IVMP monotherapy following a relapse could be attributed to prior use of immunosuppressive treatments (32). After undergoing three PLEX sessions, the patient’s condition stabilized but did not fully remit. While there is no definitive criterion guiding the initiation of PLEX, the unpredictable nature of NMOSD relapses, potential for severe disability accumulation, and the limited efficacy of IVMP monotherapy generally warrant early PLEX initiation, about 40% likelihood if started within 2 days of disease onset, dropping to 0% if initiated after 20 days (33, 34). Therefore, timely initiation of PLEX is crucial, as even a delay of 5 days can adversely affect disability outcomes (34, 35).

The involvement of B cells in the pathogenesis of SS has been unequivocally demonstrated. The high activity of B cells can be reflected by various indicators, including the presence of autoantibodies such as rheumatoid factor, SS-A/Ro, and SS-B/La, as well as the elevated levels of serum immunoglobulins and B-cell activating factors (36). Additionally, compared to other autoimmune diseases such as SLE and RA, patients with SS exhibit a significantly increased risk of developing B-cell lymphomas during the course of the disease (37).

Rituximab is a monoclonal antibody that targets CD20, which is expressed on the surface of almost all B cells. One of the first small scale randomized double-blind controlled trials reported on the unstimulated whole salivary flow rate and stimulated whole salivary flow rate in 30 SS patients with significant improvement in the rituximab group compared with placebo (38). Most studies used the Schirmer’s test to assess ocular gland function. The study by Carubbi et al. showed significant improvement of the Schirmer’s test after treatment with rituximab (39). Sandrine et al. reported an improvement of the salivary gland echostructure with rituximab (40) whereas Fisher et al. found that rituximab resulted in improvement of the total ultrasound score compared with placebo in SS patients (41). Although the role of rituximab in the treatment of SS remains to be determined, given the central role of B-cell in the pathogenesis of SS, targeted B-cell therapy is certainly a reasonable treatment to consider.

Inebilizumab, a humanized IgG1κ monoclonal antibody targeting CD19, plays a crucial role in including B-cell depletion through both antibody-dependent and complement-dependent cytotoxicity. Unlike CD20, CD19 is expressed on a broader spectrum of B cells, including precursor B cells, plasmablasts, and plasma cells, the latter being the primary producers of autoantibody. This characteristic is particularly relevant in the context of antibody-mediated autoimmune diseases such as NMOSD, SS, and pancytopenia. A notable clinical trial involving 230 NMOSD patients, with 92% being AQP4-IgG seropositive (174 receiving inebilizumab and 56 on placebo), was halted early due to the clear demonstration of inebilizumab’s efficacy (42). This efficacy was further substantiated in subsequent *post-hoc* open-label analyses (43–46). The most common side effects reported were infusion-associated reactions and urinary tract infections (42). Thus, inebilizumab was selected after comprehensive consideration. As expected, inebilizumab treatment not only significantly improved NMO symptoms, but also had a therapeutic effect on SS and pancytopenia. This case opens new avenues for the clinical application of inebilizumab.

However, the limitations of this study cannot be overlooked. During the follow-up period from May 6th to the present, there was no adverse effect and no relapse or exacerbation of the disease was observed. But, we have yet to obtain objective evidence such as laboratory tests and MRI imaging. Nonetheless, we will persist in conducting follow-ups to acquire clinical examination results from the patient and document the recurrence cycle.

## 4 Conclusion

This case report represents a significant finding that inebilizumab in AQP4-IgG seropositive NMOSD patient not only improves symptoms of NMOSD and reduces disease relapses but also effectively alleviates symptoms of concurrent SS and pancytopenia. In the cases of AQP4-IgG seropositive NMOSD who have recurrent episodes and are comorbid with other autoimmune disorders, inebilizumab may be a good choice.

## Data availability statement

The datasets presented in this article are not readily available because of ethical and privacy restrictions. Requests to access the datasets should be directed to the corresponding authors.

## Ethics statement

Ethical review and approval was not required for the study on human participants in accordance with the local legislation and institutional requirements. Written informed consent from the patients/participants or patients/participants' legal guardian/next of kin was not required to participate in this study in accordance with the national legislation and the institutional requirements. Written informed consent was obtained from the individual(s) for the publication of any potentially identifiable images or data included in this article.

## Author contributions

SL: Writing – original draft, Formal analysis, Data curation, Conceptualization. YG: Writing – original draft, Data curation. YH: Writing – review & editing, Formal analysis, Data curation. ZZ: Writing – review & editing, Project administration, Funding acquisition, Data curation.
